# *Streptococcus suis* contains multiple phase-variable methyltransferases that show a discrete lineage distribution

**DOI:** 10.1093/nar/gky913

**Published:** 2018-10-10

**Authors:** John M Atack, Lucy A Weinert, Alexander W Tucker, Asma U Husna, Thomas M Wileman, Nazreen F. Hadjirin, Ngo T Hoa, Julian Parkhill, Duncan J Maskell, Patrick J Blackall, Michael P Jennings

**Affiliations:** 1 Institute for Glycomics, Griffith University, Gold Coast, Queensland 4222, Australia; 2 Department of Veterinary Medicine, University of Cambridge, Cambridge CB3 0ES, UK; 3 Oxford University Clinical Research Unit (OUCRU), 764 Vo Van Kiet, Quan 5, Ho Chi Minh City, Viet Nam, and Nuffield Department of Medicine, University of Oxford, Oxford OX3 7BN, UK; 4 Wellcome Sanger Institute, Cambridge CB10 1SA, UK; 5 Queensland Alliance for Agriculture and Food Innovation, The University of Queensland, St Lucia, Queensland 4072, Australia

## Abstract

*Streptococcus suis* is a major pathogen of swine, responsible for a number of chronic and acute infections, and is also emerging as a major zoonotic pathogen, particularly in South-East Asia. Our study of a diverse population of *S. suis* shows that this organism contains both Type I and Type III phase-variable methyltransferases. In all previous examples, phase-variation of methyltransferases results in genome wide methylation differences, and results in differential regulation of multiple genes, a system known as the phasevarion (phase-variable regulon). We hypothesized that each variant in the Type I and Type III systems encoded a methyltransferase with a unique specificity, and could therefore control a distinct phasevarion, either by recombination-driven shuffling between different specificities (Type I) or by biphasic on-off switching via simple sequence repeats (Type III). Here, we present the identification of the target specificities for each Type III allelic variant from *S. suis* using single-molecule, real-time methylome analysis. We demonstrate phase-variation is occurring in both Type I and Type III methyltransferases, and show a distinct association between methyltransferase type and presence, and population clades. In addition, we show that the phase-variable Type I methyltransferase was likely acquired at the origin of a highly virulent zoonotic sub-population.

## INTRODUCTION

*Streptococcus suis* is a major veterinary pathogen, causing a range of chronic and severe diseases in pigs such as arthritis and septicaemia (1). These diseases are responsible for major economic losses to agriculture globally. *Streptococcus suis* is also a major cause of zoonotically acquired bacterial meningitis in humans, particularly in South-East Asia (2) where it is an endemic public health concern, with high mortality rates associated with a Streptococcal septic/toxic shock like syndrome (3). Although human-to-human transmission appears rare, an increasing incidence of zoonotically acquired disease is associated with increasing human populations and intense agricultural practices (4). Risk factors for *S. suis* infection in humans appear to be prolonged contact with pigs, and consumption of undercooked pork and pork-products (5). The majority of human cases of *S. suis* infection appear to be associated with serogroup 2, although other serogroups have been reported to cause disease (6–8). A recent study has shown that there are no consistent genomic differences between strains that cause disease in humans versus those that cause disease in pigs, and that strains isolated from humans are confined to a single lineage that emerged in the 1920s when pig production was intensified (9).

Many host adapted bacterial pathogens contain phase-variable genes. Phase variation is the random on-off switching of gene expression. This process often occurs by the variability in simple sequence repeats (SSRs) within a gene or promoter, or by the shuffling of sequences by homologous recombination between expressed and silent loci via inverted repeats (IRs) present in these loci (10). Phase variation is often associated with genes that encode bacterial surface factors such as adhesins (11–13), pili (14), flagella (15) iron-acquisition proteins (16,17), and lipo-oligosaccharide (18,19). However, many host-adapted bacterial pathogens contain phase-variable methyltransferases, associated with restriction-modification (R-M) systems, that are able to phase-vary (20,21), and are involved in regulation of multiple genes via epigenetic mechanisms, in a system known as the phasevarion (for **phase**-**vari**able regul**on**) (22). These systems control gene regulation in a number of important human pathogens, including non-typeable *Haemophilus influenzae* (23), *Moraxella catarrhalis* (24), pathogenic Neisseria (25) and *Streptococcus pneumoniae* (26). All these organisms, with the exception of *S. pneumoniae*, contain Type III methyltransferases that contain SSRs, with variation in SSR number leading to biphasic on-off switching of methyltransferase expression. *Streptococcus pneumoniae* is the first example of a Gram-positive organism containing a phasevarion, and also the first example of a phasevarion controlled by a Type I methyltransferase (26). In contrast to the biphasic on/off phasevarions previously characterized in Type III methyltransferases, shuffling of specificity domains between expressed and silent loci leads to six different methyltransferase specificities. This results in a six-phase switch, and consequently six different phenotypic states within a pneumococcal population (26). All phasevarions characterized to date regulate putative vaccine candidates and virulence factors (22–27). This has clear implications in the development of stable vaccines and treatment strategies: it is only possible to define the stably expressed protein profile in organisms containing phasevarions by studying the genes regulated by randomly switching methyltransferases in these organisms. Here we report the identification and analysis of multiple phase-variable methyltransferases in *S. suis*.

## MATERIALS AND METHODS

### Bacterial strains and growth conditions

*Streptococcus suis* isolates were grown on Brain-Heart Infusion (BHI) agar or Sheep-blood agar (Oxoid), with liquid cultures grown in Todd-Hewitt broth containing 0.2% yeast extract (Oxoid) at 37°C. *Escherichia coli* DH5α and BL21 were grown in Luria–Bertani media supplemented with 100 μg/ml ampicillin where appropriate, at 37°C with 200 rpm shaking. Prototype *S. suis* strains used in this study are detailed in Table 1.

**Table 1.** Prototype strains used in this study

*S. suis* strain	Phase-variable methyltransferase locus	Allelic variant	Genbank locus tag (accession number)	Additional information
P1/7^§^	Type I	allele B	SSU1271-SSU1274 (AM946016)	
S735	Type I	allele B	YYK_06100- YYK_06115 (CP003736)	population also contains minor variants A, C, D (Figure 1)
T15^§^	Type III	*modS1*	T15_1305 (CP006246.1)	*modS1* locus first observed in strain T15*
LSS89^#^	Type III	*modS1*	(FIJA01000010.1)	
SS1028^#^	Type III	*modS2*	(FIJX01000003.1)	
LSS66	Type III	*modS2*	(FIHP01000004.1)	also contains two Type I signatures and Dam (Table 3)
YS77^#^	Type III	*modS3*	(ALNH01000046.1)	

*described in reference (39).

^#^gene was cloned from these strains and the methyltransferase expressed heterologously in *E. coli*, as described in ‘Material and Methods’ section.

^§^locus first identified in these strains/sequences used to search custom database.

### Molecular biology

Standard molecular biology techniques were used throughout (28). Genomic DNA was prepared using the Sigma GenElute bacterial genomic DNA kit according to manufacturer's instructions. Plasmid DNA for SMRT sequencing was prepared using the Promega Wizard mini prep kit according to manufacturer's instructions (Promega). Oligonucleotide primers were purchased from Integrated DNA Technologies, and are detailed in Table 2. Polymerase chain reaction (PCR) was carried out using GoTaq polymerase (Promega), or KOD HotStart polymerase (Merck) according to manufacturer's instructions. Restriction enzymes were purchased from New England Biolabs, and used according to manufacturer’s instructions. DNA fragment length analysis was carried out at the Griffith University DNA Sequencing Facility (GUDSF; Brisbane, Australia), or the Australian Genome Research Facility (AGRF; Brisbane, Australia). Sequencing was carried out using Big Dye 3.1 (Perkin Elmer), using PCR products purified using the Qiagen PCR purification kit according to the manufacturer's instructions. Samples were analysed by GUDSF or AGRF.

**Table 2.** Oligonucleotides used in this study

Name	Sequence
Ssu-T1-For-FAM	5′-FAM-CCAAATGATGAGCCTGCAAGTGAAC
Ssu-T1-Rev	5′-CCTTAATCCCAACCTGCTCTTTTAG
SsuT3-F-FAM	5′-FAM-CATCAAAAACGGCTTGACAGCC
SsuT3-R	5′-GCAATGTTGTCTGATAAAACATCTTTTG
SsuT3-oE-F	5′-AGTCAGCCATGGGGGAGCAGAAGCTTGGAGACTACACTCAAG
SsuT3-oE-R	5′-AGTCAGGGATCCCTACACCACCTTCACTTTGGTACC
RT-16S-Ssu-F	ATGGACCTGCGTTGTATTAGC
RT-16S-Ssu-R	CATTGCCGAAGATTCCCTAC
RT-SsuT1-5′_1-F	GGT TTT GGA GAT ACA CTT CTC C
RT-SsuT1-5′_2-F	CCA TTC GTA GCA CAA ACT CAA C
RT-SsuT1-3′_1-R	GAA GAG TCC AAT TTC TCG GAA TC
RT-SsuT1-3′_2-R	GTA GCA CCA AGA ACT AGG AAG

*16s rRNA primer pair sequences from (51).

### Type I allele quantification

The variant alleles of *hsdS* in *S. suis* strain S735 were quantified utilising FAM labelled PCR coupled to a restriction digest in a method similar to that used previously for *S. pneumoniae* (26). The whole *hsdS* locus was PCR amplified (3.02 kb) from extracted genomic DNA prepared from an entire plate of *S. suis* strain S735 grown overnight on BHI agar. PCR was carried out using GoTaq DNA polymerase (Promega) according to the manufacturer’s instructions, and utilising primers that bind outside the area of recombination (Ssu-T1-For-FAM and Ssu-T1-Rev). The PCR products were then digested in a double-digest with DraI and NciI (New England Biolabs). This digestion was predicted to yield different sized FAM-labelled fragments for each of the variant forms. The pool of restriction fragments was run on an ABI prism Gene Analyser (Life Technologies). The area of the peak given by each labelled fragment, each corresponding to the prevalence of one of the variant forms, was quantified using Peak Scanner v1.0.

### Type III allele quantification

Fragment length analysis of the phase-variable Type III methyltransferase repeat tract was conducted using the fluorescently labelled forward primer SsuT3-F-FAM and the reverse primer SsuT3-R, and fragments were analysed by the Australian Genome Research Facility (AGRF, Brisbane, Australia). PCR was carried out using cell suspensions using the relevant *S. suis* strain grown over-night on BHI agar.

### RNA preparation and semi-quantitative RT-PCR

**Figure 1.**
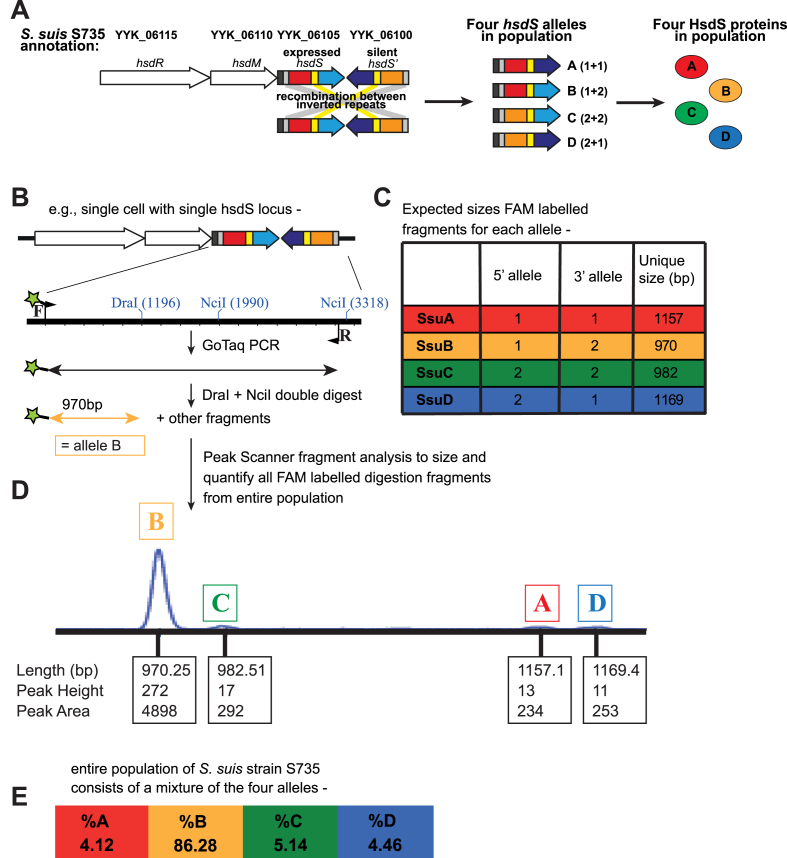
Demonstration that *Streptococcus suis* contains a Type I *hsd* locus that is able to phase-vary. (**A**) Illustration of the Type I *hsd* R-M locus from *S. suis* strain S735 containing duplicate, variable hsdS specificity loci that contain IRs (yellow and grey boxes). By shuffling between variable 5′ (red and orange regions) and 3′ (blue and purple) TRDs, four unique *hsdS* genes can be encoded at the *hsdS* expressed locus immediately downstream of the *hsdM* gene. This results in the expression of four unique allelic variants of the HsdS protein, all of which are predicted to lead to different methyltransferase specificities; (**B**) Illustration of FAM-labelled PCR assay used to type and quantify *hsdS* alleles present in the expressed locus in the *S. suis* population, with FAM label illustrated as green star on forward primer, and (**C**) predicted sizes of each allelic variant based on *in silico* analysis. (**D**) Following PCR and restriction digest, FAM-labelled fragments are separated and quantified using a Gene Analyser (Life Technologies) and quantified used Peak Scanner software in order to determine the percentage of each allele present in a *S. suis* population; (**E**) quantification of *hsdS* alleles A–D present in a population of *S. suis* strain S735 following Peak Scanner analysis.

Whole cell RNA preps were made from *S. suis* strain S735 using cells grown overnight on BHI plates, so as to replicate the growth conditions used for the Type I allele quantification assay (above; Figure 1). RNA was prepared using Trizol reagent (Thermo-Fisher) according to the manufacturer’s instructions. Prior to reverse transcription (RT), RNA was treated with heat-labile dsDNAse (Thermo Fisher catalogue number EN0771) according to manufacturer’s instructions, and used directly in the RT reaction. cDNA was synthesized from DNAse-treated RNA using Protoscript II Reverse Transcriptase (New England Biolabs) according to manufacturer’s instructions. Negative controls were carried out by omitting the RT from the cDNA synthesis reaction. Semi-quantitative PCR was carried out in a multiplex reaction using GoTaq DNA polymerase (Promega) in a standard PCR reaction with primers specific for 16s rRNA, and one of four Type I allele-specific primer pairs (alleles A, B, C or D; Table 2). 16s rRNA primers have been described previously (51).

### Cloning and over-expression of Type III methyltransferase variants

PCR products for cloning into the NcoI/BamHI site of pET15b cloning vector (Merck) were prepared using KOD Hot-start DNA polymerase (Merck) according to manufacturer's instructions. Primers specific for the Type III system were designed to clone the gene containing only one GAGCA repeat so as to lock-on the methyltransferase (SsuT3-oE-F and SsuT3-oE-R). We cloned the three unique *mod* alleles from *S. suis* strains LSS89 (*modS1*), SS1028 (*modS2*) and YS77 (*modS3*). A vector containing an non-methyltransferase, *siaB*, used previously (23) served as a non-methylating control. Over-expression of each protein was carried out using *E. coli* BL21 cells, which were induced by the addition of Isopropyl β-D-1-thiogalactopyranoside (IPTG) to a final concentration of 0.5 mM overnight at 37°C with shaking at 200 rpm.

### Single-molecule, real-time (SMRT) sequencing and methylome analysis

Mini-preps from *E. coli* cells expressing each methyltransferase and a negative control expressing an the non-methyltransferase *siaB*, described previously (23) were prepared using the Promega wizard plasmid mini kit according to the manufacturer’s instructions. Genomic DNA from *S. suis* strain LSS66 was prepared from an overnight culture in Todd Hewitt broth (Oxoid) +0.2% Yeast Extract (Fisher Scientific) and cells lysed according to the Genomic DNA Handbook (Qiagen) using 100 mg/ml Lysozyme (Sigma) supplemented with 250 units/ml Mutanolysin (Sigma). High-molecular-weight genomic DNA was isolated using Genomic-tip 100/G columns (Qiagen) according to the manufacturer's instructions and DNA resuspended in 10 mM Tris–Cl, pH 8.5 (Qiagen) overnight at 4°C. A phenol:chloroform:isoamylalcohol (Sigma) clean-up was then performed before finally resuspending DNA in 10 mM Tris–Cl, pH 8.5 (Qiagen) overnight at 4°C. SMRT sequencing and methylome analysis was carried out as previously (29,30). Briefly, DNA was sheared to an average length of ∼0.5–2kb (plasmids) or 5–10 kb (genomic DNA) using g-TUBEs (Covaris, Woburn, MA, USA) and SMRTbell template sequencing libraries were prepared using sheared DNA. DNA was end repaired, then ligated to hairpin adapters. Incompletely formed SMRTbell templates were degraded with a combination of Exonuclease III (New England Biolabs; Ipswich, MA, USA) and Exonuclease VII (USB; Cleveland, OH, USA). Primer was annealed and samples were sequenced on the PacBio Sequel system (Menlo Park, CA, USA) using standard protocols for long insert libraries. SMRT sequencing and methylome analysis was carried out at the Yale Centre for Genome Analysis (plasmid vectors) or the Wellcome Sanger Institute (genomic DNA; Cambridge, UK). Initial methylation analysis of DNA from strain L66 using the standard SMRT analysis pipeline showed a very high noise level, so it was reanalysed using whole-genome amplified DNA as a methylation-negative control.

### Bioinformatic analysis

The strain collection used to determine the Type I and Type III methyltransferase distribution was described previously (9,31). This collection comprises of isolates from different countries (UK, Vietnam, China, the Netherlands) and are associated with different clinical phenotypes of pigs. Non-clinical isolates come from the tonsils and upper-respiratory tract of pigs without signs of *S. suis* disease, respiratory isolates come from the lungs of pigs with signs of pneumonia, and systemic isolates come from the blood, brain or joints of a pig with signs of systemic disease. Sequencing was performed after isolation on Columbia agar (Oxoid Ltd. Basingstoke, UK) containing 5% (v/v) sheep blood (TCS biosciences Ltd., Bucks, UK) as described previously (9). We took the entire *hsd* region from *S. suis* P1/7 (Genbank locus tags SSU1271-SSU1274) and the *mod* locus from *S. suis* T15 (T15_1305) and used blastn against a custom blast database containing the collection and published complete genomes (Supplementary Data 1) to score presence and absence. A core genome of the collection was produced by extracting the protein sequences from *S. suis* strain P1/7 and using these as a tblastn query against a custom blast database containing the collection and published complete genomes (Supplementary Data 1). All sequences that showed a minimum of 80% amino acid identity across 80% of the length of the protein to the *S. suis* P1/7 (Genbank ID: AM946016) and which were present in every isolate were included in the core genome. A neighbour-joining tree of *S. suis* was made from this core genome using a pairwise distance matrix created with the ‘K80’ model (32) of sequence evolution in the package ape in R (33). Bayesian populations (BAPS groups) were taken from Weinert *et al.* (9). The neighbour joining tree with associated meta data presented in Figure 3 was created using the interactive Tree of Life (34).

## RESULTS

### Distribution and variation in the phase-variable methyltransferases of *S. suis*

**Figure 2.**
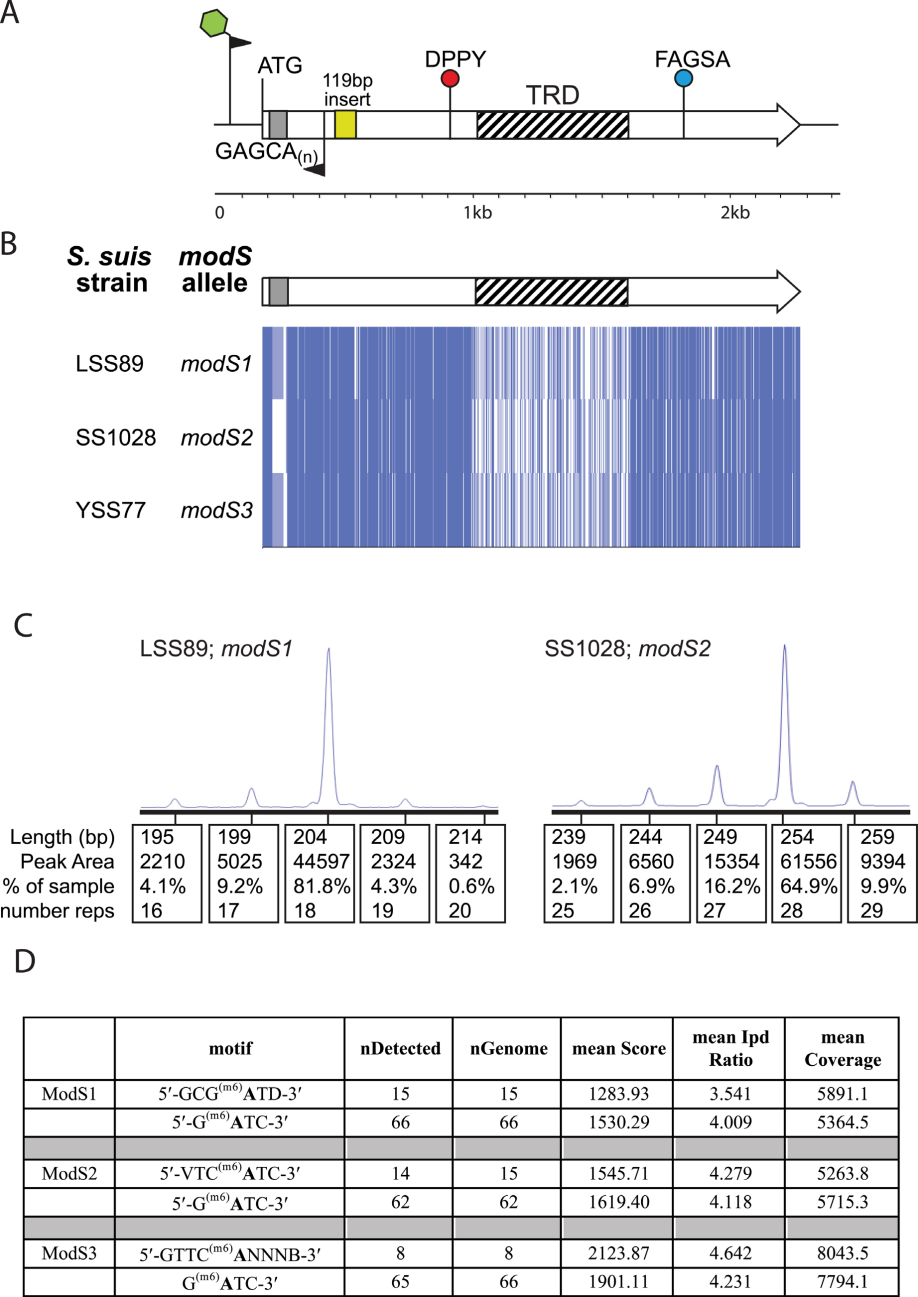
*Streptococcus suis* contains three allelic variants of the new phase-variable Type III *modS* gene. (**A**) Illustration of the Type III *mod* gene, *modS*, present in multiple strains of *S. suis*, showing the location of key features; DPPY—catalytic motif; FXGXG—AdoMet substrate binding motif; repeat tract at 5′ end of gene shown by a grey box and GAGCA_(n)_; 119 bp ‘insert’ sequence shown by yellow box; location of FAM-labelled primer pair used to carry out fragment length analysis (results in panel **C**) shown, with FAM label illustrated as green star on forward primer; (**B**) alignment of the three *modS* alleles identified in this study, alleles *modS1, modS2* and *modS3*, from *S. suis* strains LSS89, SS1028 and YS77, respectively. Alignments were carried out using ClustalW, and visualized in JalView overview feature; (C) Fragment length analysis PCR and quantification of fragment sizes using a Gene Analyser (Life Technologies) and quantified used Peak Scanner software. Percentage of each peak area indicates the percentage of the total bacterial population containing the number of repeats indicated by that particular peak size (bp); and (**D**) SMRT sequencing and methylome analysis results of plasmid vectors from *Escherichia coli* BL21 strains over-expressing one of the three ModS allelic variants, demonstrating the methyltransferase specificity for ModS1 (5′-GCG^(m6)^**A**TD-3′), ModS2 (5′-VTC^(m6)^**A**TC-3′) and ModS3 (5′-GTTC^(m6)^**A**NNNB-3′). Dam methylation (5′-G^(m6)^**A**TC-3′) from the same vector is shown underneath the respective ModS data in the table. nDetected = number of sites called as methylated in each plasmid vector; nGenome = number of sites present in each plasmid vector; IPD = interpulse duration. Only motifs that had an IPD > 3.5 are determined to be significant as methylated. Nucleotide codes: *N* = any nucleotide; B = G, C or T; D = A, G or T; V = A, G or C.

We examined a large and diverse collection of *S. suis* genome sequences that has been described previously (*n* = 377) (9). We also examined all those strains in Genbank with annotated genomes (*n* = 16; therefore total number of strains = 393). This analysis examined the presence of both phase-variable Type I and Type III methyltransferases within *S. suis* genomes. Of these 393 *S. suis* strains, 262 strains contained a phase-variable Type I system, characterized by duplicate, variable *hsdS* loci containing IRs (see Figure 1A) (35) and 41 strains contained a phase-variable Type III *mod* gene, characterized by locus located SSRs (see Figure 2A) (20). Ninety strains contained neither a Type I nor a Type III phase-variable methyltransferase. All strain data is presented in Supplementary Data 1.

### Most *S. suis* strains contain a Type I methyltransferase with duplicated hsdS specificity loci

Type I R-M systems have three subunits, containing restriction (HsdR), methyltransferase (HsdM) and specificity (HsdS) components (Figure 1A), with the HsdS subunits dictating where the restriction enzymes methylate and cleave DNA (36). Each complete HsdS protein is made up of two half-target recognition domains (TRDs), each contributing half to the overall specificity. In our previous studies with *S. pneumoniae* (26), we demonstrated that duplicated, inverted silent *hsdS* loci provide a repository for alternate specificity proteins that can be generated by recombination with the expressed *hsdS* locus. Upon examination of Type I R-M systems in *S. suis*, we observed that many contained a Type I R-M system with duplicated *hsdS* loci (Figure 1), and that the sequence of the expressed *hsdS* gene, located immediately downstream of the *hsdM* and *hsdR* components, showed a variability in sequence when different strains of *S. suis* were compared. Examination of these loci showed the presence of IRs that would allow homologous recombination to occur, resulting in shuffling of the TRDs between the expressed *hsdS* locus and the downstream silent *hsdS*′ (Figure 1A). Analysis of these *hsdS* sequences showed that there were two variable 5′-TRD sequences, and two variable 3′-TRD sequences, meaning four unique *hsdS* genes can be produced, which we have named *hsdS* alleles A-D (Figure 1A; allele A contains 5′-TRD-1 and 3′-TRD-1; allele B contains 5′-TRD-1 and 3′-TRD-2; allele C contains 5′-TRD-2 and 3′-TRD-2; and allele D contains 5′-TRD-2 and 3′-TRD-1). Upon examination of the sequence of the 262 strains containing a Type I locus with duplicated *hsdS* genes, we discovered that the majority (260/262) of Type I alleles present in the expressed *hsdS* locus of the Type I system consist of 5′-TRD-1 and 3′-TRD-2, which we have designated allele B. Only two strains contain allele A, consisting of 5′-TRD-1 and 3′-TRD-1. No strains contained allele C (5′-TRD-2 and 3′-TRD-2) or allele D (5′-TRD-2 and 3′-TRD-1) in the expressed *hsdS* locus immediately downstream of the *hsdM* gene. However, this apparent lack of either allele C or allele D in the expressed *hsdS* locus of *S. suis* genomes could be a result of the genome annotation process – minority alleles do exist in a population but this locus will only be annotated as the majority allele in the final sequence annotation. In order to investigate if these duplicate, ‘tail-to-tail’ *hsdS* genes could undergo homologous recombination to produce variable *hsdS* sequences in the expressed locus immediately downstream of the *hsdM* gene, even in the absence of a locus-associated recombinase, we designed a fluorescent PCR reaction coupled to a restriction digest in order to type and quantify the alleles present within a population of *S. suis* (Figure 1B–E). Using *S. suis* strain S735 (Type I locus YYK_06100- YYK_06115; Table 1), this assay shows that the majority of the population (86.27%) of this strain contains *hsdS* allele B (5′-TRD-1 and 3′-TRD-2) in the expressed *hsdS* locus (Figure 1D and E), confirming the annotated genome sequence (accession number CP003736.1). However, 4.12% of the population contains *hsdS* allele A (5′-TRD-1 and 3′-TRD-1), 5.14% of the population *hsdS* allele C (5′-TRD-2 and 3′-TRD-2) (Figure 1D and E), and 4.46% of the population *hsdS* allele D (5′-TRD-2 and 3′-TRD-1) (Figure 1D and E). To demonstrate that all four of these encoded *hsdS* alleles are expressed, we carried out semi-quantitative RT-PCR to demonstrate the presence of RNA encoding all four alleles within a population of *S. suis*. Using RNA prepared from strain S735, we show that the majority of the population express RNA encoding allele B (Supplementary Figure S1), and that there is also the presence of RNA encoding each of the three minor variants (alleles A, C and D) which we show are present in the genomic DNA isolated from strain S735 grown under the same conditions (Figure 1 and Supplementary Figure S1). This demonstrates that these encoded minor variants are expressed within a population. We have also carried out analysis on cells grown in liquid media (BHI broth) and observe similar allele distributions as those shown in Figure 1 (data not shown).

Our examination of genome sequences of *S. suis* also demonstrated the presence of two additional Type I R-M loci (YYK_03085-YYK_03100 and YYK_07615-YYK_07625 in strain S735). Previous in depth analysis of Type I R-M systems in *S. suis* has shown that homologues of these loci are distributed across the majority of *S. suis* strains (37). This work also demonstrated the presence of the phase-variable Type I R-M system studied here, but did not show phase-variation within the population of an individual strain as we have.

### Multiple alleles of a phase-variable Type III methyltransferase are present in *S. suis*

Examination of the 41 strains that contained a phase-variable Type III *mod* gene showed the presence of two unique coding sequences, encoding two distinct Type III methyltransferase enzymes. Sequence alignment of these genes demonstrated that they are highly conserved in their 5′ and 3′ regions (>95% nucleotide identity), with high diversity observed in the central TRD (<25% nucleotide identity), which dictates the DNA sequence to be methylated (38) (Figure 2). Therefore, these two genes are allelic variants of the same gene, rather than two separate genes. Of the 41 genes containing a Type III *mod* gene with 5′-GAGCA_(n)_ repeats, 29 contain an identical gene to the one we identified in a single strain of *S. suis*, strain T15, in our systematic study of all SSR-containing Type III *mod* genes in REBASE (39). We chose strain LSS89 as our prototype strain of *S. suis* containing this allele. The remaining 12 strains contained a different allele of the same gene (Figure 2B). We chose strain SS1028 as our prototype strain of *S. suis* containing this allele. In order to examine if any other new alleles of this newly identified phase-variable methyltransferase were present in *S. suis*, but not captured in our strain collection, we performed a BLAST search of all whole genome shotgun contigs present in Genbank. This analysis identified a third allele, present in a single *S. suis* isolate, strain YS77 (Figure 2B), isolated in China. In order to demonstrate that phase-variation of the GAGCA_(n)_ repeat tract occurs in these genes, leading to variable expression of the *mod* gene, we performed FAM-labelled PCR coupled to fragment length analysis, over the repeat tract of the alleles identified in strains LSS89 and SS1028, as we have used previously to characterize and quantify expression of phase-variable genes (18,23). This analysis demonstrated both a difference in repeat numbers present in these genes (Figure 2C; major peaks of 18 repeats in LSS89, 28 repeats in SS1028), and minor populations of each strain containing variable numbers of repeats around the major peak size, highly indicative of ON-OFF switching *within* the population of each individual strain (Figure 2C). We also observe a high variability in GAGCA_(n)_ repeat tract length between each of the 41 Type III *mod* genes in our *S. suis* strain collection; this is also an excellent indication that phase-variable expression of this gene is occurring (Supplementary Data 1), as this phenomenon is seen in every other example of a Type III *mod* gene that has been demonstrated to be phase-variable (20,39).

In line with our previous studies (23,25,27,40) we hypothesized that the three identified *mod* alleles in *S. suis* methylate different DNA target sequences, and therefore control different phasevarions, as they all encode for different TRDs. Single-molecule, real-time (SMRT) sequencing and methylome analysis using plasmid DNA isolated from *E. coli* BL21 over-expressing each allelic variant demonstrated that these three alleles all methylate different DNA target sequences (Figure 2D). The allele from strain LSS89, identical to that initially identified in strain T15, methylates the sequence 5′-GCG^(m6)^**A**TD-3′ (Figure 2D). The second allele identified in our *S. suis* strain collection (from strain SS1028) methylates the sequence 5′-VTC^(m6)^**A**TC-3′ (Figure 2D). The third allele, from strain YS77, methylates the sequence 5′-GTTC^(m6)^**A**NNNB-3′ (Figure 2D). Dam methyltransferase activity (5′-G^(m6)^**A**TC-3′) occurs in all three vectors, as would be expected in DNA isolated from *E. coli* (Figure 2D). We could detect only Dam methylation in our non-methylating control (not shown). In order to align naming conventions with current phase-variable Type III methyltransferases, we propose naming these alleles *modS1* (LSS89), *modS2* (SS1028) and *modS3* (YS77) (39), with systematic names based on REBASE conventions (41) of S.Ssu89I (ModS1), S.Ssu1028I (ModS2) and S.Ssu77I (ModS3). We also carried out PacBio SMRT sequencing and methylome analysis using genomic DNA prepared from *S. suis* strain LSS66 containing allele *modS2*. Our analysis of the genome sequence generated previously (9) showed that this strain contained GAGCA_[6]_ in the *modS2* ORF, which would lead to the gene being in frame (ON). This low number of repeats is highly stable, with low rates of phase-variation predicted based on previous analyses of SSR length and phase-variation rate (42–44). *modS2* was the only annotated Type III *mod* gene present in the genome of strain LSS66, so we predicted that any Type III methyltransferase signatures detected could be attributed to ModS2 methylation. Our methylome analysis detected a single Type III signature, 5′-VTC^(m6)^**A**TC-3′ (Table 3), identical to that identified above using plasmid DNA isolated from a strain over-expressing ModS2 from a different strain of *S. suis*, SS1028 (Figure 2D). This not only confirmed the sequence recognized and methylated by ModS2, but also showed that the same allele has the same specificity in different strains of *S. suis*, and therefore is highly likely to regulate the same phasevarion. The LSS66 methylome demonstrated the presence of two Type I motifs in this strain (Table 3), which we predict to be the sequence specificities of the two non-phase-variable Type I loci previously reported to be present in all strains of *S. suis* (37), but we cannot confidently assign these signatures to particular methyltransferases without significant additional analysis, which is outside the scope of the current work. An examination of REBASE (41) shows that these two Type I motifs have not previously been reported. All methylome data from strain LSS66 is presented in Table 3.

**Table 3.** PacBio SMRT sequencing and methylome data from *S. suis* strain LSS66

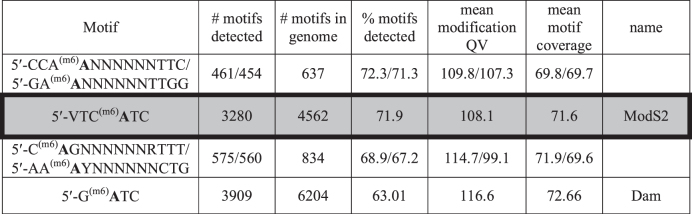

**Figure 3.**
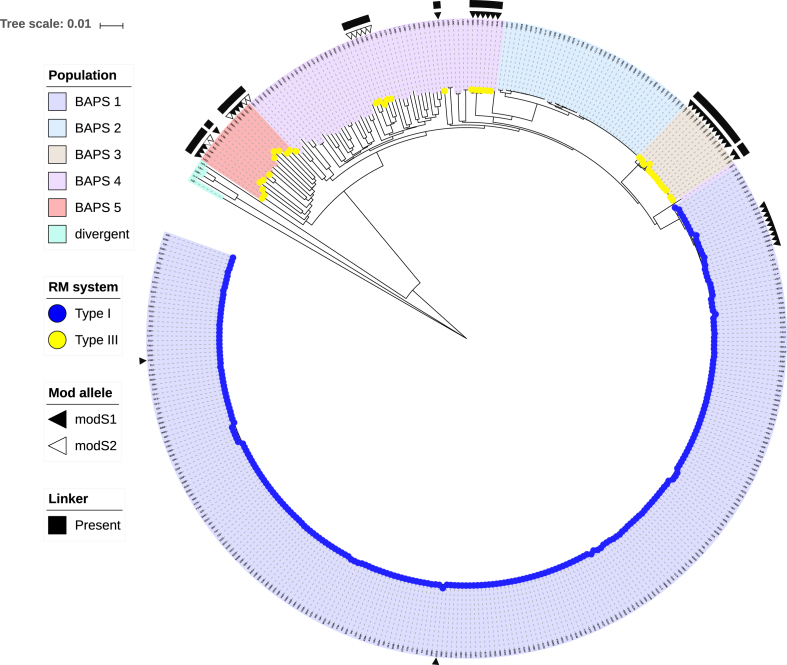
The distribution of the phase-variable Type I and Type III methyltransferases in *Streptococcus suis*. A neighbour joining tree showing the genetic distance between isolates based on all sites in a core genome of *S. suis* taken from Weinert *et al.* (9). BAPS are represented by coloured clades and the presence of the phase-variable Type I methyltransferase (blue) and the presence of the phase-variable Type III methyltransferase (yellow) are indicated by coloured tips. The Type I methyltransferase is restricted to BAPS population 1, which has been shown to be a virulent zoonotic population (9). The Type III methyltransferase (*modS*) is more interspersed, being found in three different populations, with two populations containing a mixture of the *modS1* (filled triangle) and *modS2* (empty triangle) alleles (shown as the second column). The Type III methyltransferase with *modS1* allele is also found within population 1 but all isolates lack the 119 bp ‘linker’ sequence (presence shown as filled square in the first column). The scale bar indicates the genetic distance (number of substitutions per site).

An interesting finding from the analysis of our *S. suis* strain collection was that strains containing a Type I locus with duplicated *hsdS* genes also contained a GAGCA_(n)_ immediately upstream of a putative Type III *mod* gene. However, closer examination of this region showed that a functional Type III *mod* gene containing SSR’s only occurred if a unique 119 bp sequence was present between the SSRs and the putative *mod* gene (shown schematically in Figure 2A), linking the two regions into a single, continuous open reading frame. Where this 119 bp ‘linker’ sequence was present, resulting in a phase-variable *mod* gene, those strains did not contain a Type I locus with duplicated *hsdS* genes. In those strains containing duplicated *hsdS* genes, and by implication a phase-variable Type I methyltransferase, GAGCA_(n)_ repeats were present, sometimes in very high numbers (Supplementary Data 1), but the 119 bp ‘linker’ sequence was not (filled square in Figure 3). Therefore, it appears that a single strain of *S. suis* cannot contain both phase-variable Type I and Type III methyltransferases (yellow and blue coloured tips in Figure 3).

### A clear segregation exists between phase-variable methyltransferases and population structure in *S. suis*

Our previous studies with *S. suis* defined five genetic lineages (BAPS) based on a Bayesian clustering method (9) as well as an additional ‘divergent’ lineage of *S. suis* (Baig *et al.* (15)). Upon examination of BAPS group (9) and the presence of each of the distinct types of methyltransferase, a clear segregation was evident (Figure 3). The phase-variable Type I system was only present in BAPS group 1, with the majority of these being systemic isolates (Table 4), and serotype 2, and containing all the *S. suis* isolates from humans (Supplementary Data 1). All members of this virulent zoonotic group contained the Type I system indicating it was acquired at the origin of the BAPS group 1 lineage. Phase-variable Type III *mod* genes were found in variable numbers of isolates from BAPS group 3, 4 and 5, and within a variety of serotypes, suggesting that it has been independently acquired and/or lost multiple times. In contrast to the Type I system, Table 4 shows that the Type III system is under-represented in systemic isolates but over-represented in respiratory and non-clinical isolates, although further work is needed to verify if this association is robust or a reflection of sampling bias (the most obvious bias being the oversampling of isolates from the virulent zoonotic population BAPS group 1). The majority of the isolates containing a phase-variable Type III *mod* gene are from strains isolated in the UK (39/41; 95%). Only strains T15 (Netherlands) and R61 (China) were not isolated in the UK. Interestingly, all strains from BAPS group 2 contained neither phase-variable methyltransferase, with a large proportion of BAPS group 4 (44/57; 77.2%) also containing neither system (Figure 3 and Supplementary Data 1). In summary, we predict that the phase-variable Type I methyltransferase arose once at the origin of the virulent zoonotic population of *S. suis* (BAPS group 1), but that the phase-variable Type III methyltransferase has either been independently acquired at least three times in different populations, or alternatively been lost multiple times from isolates of *S. suis*.

**Table 4.** The association between phase-variable Type I and Type III methyltransferases and the clinical phenotype of *S. suis* isolates

	Clinical phenotype				
	Systemic	Respiratory	Non-clinical	Not known	Total
Phase-variable Type I	208	8	34	12	262
Phase-variable Type III	3	10	20	8	41
neither	13	23	41	13	90
All isolates	224	41	95	33	393

## DISCUSSION

Phase variation of single genes by bacterial pathogens is well studied, and is typically associated with surface factors (11,12,14,18,19). When a bacterial strain contains several individual phase-variable genes, the population as a whole will contain a broad and diverse array of phenotypes (10). The discovery of phasevarions in a diverse array of bacterial species further compounds the problem of phenotypic diversity from a vaccine and therapeutic development point of view. Whilst it is easy to identify individual phase-variable genes by *in silico* analysis due to the genetic features associated with them (SSRs, IRs), and thus exclude them as stably expressed antigens, the identification of members of a phasevarion can only be accomplished by studying the strains that contain phase-variable methyltransferases (21,45). The first major steps in characterizing a new phasevarion is to prove phase variation occurs then to identify the target sites of each phase-variable methyltransferase, in order to identify regions that are methylated and to correlate this with an expression profile analysis of strains containing each distinct methyltransferase state: ON or OFF for phase-variable Type III methyltransferases, or each distinct HsdS expression state for phase-variable Type I methyltransferases. We have demonstrated that there are different allelic variants of *hsdS* genes present in different strains of the expressed locus of this system, and that variable SSR tract lengths exist in different strains containing a phase-variable Type III *mod* gene. To confirm that phase-variation occurs *within* a bacterial population, we demonstrated that switching of *hsdS* genes occurs within *S. suis* strain S735, by showing that in addition to the major expressed *hsdS* allele (allele B), the three minor variants (alleles A, C and D) could all be detected using our bespoke FAM-labelled PCR assay (Figure 1). For the phase-variable Type III methyltransferase, we demonstrate that variable tract lengths exist in this locus using prototype strains containing the two most common allelic variants of what we have termed *modS, modS1* (strain LSS89) and *modS2* (strain SS1028) (Figure 2C). Phase-variation by slipped-strand mis-pairing over SSRs appears to be much more prevalent in Gram-negative bacteria than in Gram-positives (46), although there are several examples of phase- and antigenic-variation in Gram-positive organisms (13,15,46–48). Our demonstration of the presence and functionality of a phase-variable Type III methyltransferase in *S. suis* is the first example of SSRs resulting in phase-variable expression of a methyltransferase in a Gram-positive organism.

The advent of PacBio SMRT sequencing technology coupled to methylome analysis has revolutionized the identification of methyltransferase specificity, and allows the analysis of the complete methylome of bacterial strains, whilst simultaneously allowing precise identification and methylation state characterization of each target site in the genome. This has allowed us to identify the methylation specificities from all three *modS* allelic variants present in *S. suis* (Figure 2D). The specificities of the four Type I *hsdS* alleles are being determined in ongoing studies addressing gene expression profile analysis of *S. suis* strain S735 expressing these alleles.

Our demonstration of a distinct lineage-specific segregation of phase-variable methyltransferases in *S. suis* is the first description of its type. The segregation we see with the phase-variable Type I and Type III methyltransferases is reminiscent of the mutually exclusive expression of the adhesins HMW and Hia in non-typeable *Haemophilus influenzae*, where it is proposed that each adhesin was acquired in separate events followed by divergence (49). In the case of phase-variable methyltransferases in *S. suis*, it is possible that too much phase-variable methylation, and consequent variation in gene expression levels is deleterious—a balance must be achieved between adaptability and amount of variability generated by epigenetic gene regulation. Here we present the first characterized example of individual bacterial strains of any species having both phase-variable Type I and Type III methyltransferases, and thereby multiple phasevarions. There have been previous reports of strains with multiple phase-variable Type III methyltrasferases in certain bacterial species: for example, *Neisseria meningitidis* can encode *modA, modB* and *modD* (50), and strains of *Helicobacter pylori* can also encode three different phase-variable Type III *mod* genes (27,39). The presence of phase-variable Type III methyltransferases has been systematically analysed in large genome collections (39), but there has been no equivalent systematic search for phase-variable Type I R-M systems. Thus, frequency of the presence of multiple phase-variable Type I and Type III methyltransferases in bacterial genomes remains to be determined. The exact individual events that have contributed to strains of *S. suis* containing only either a Type I or a Type III phase-variable methyltransferase are therefore still open to investigation since the generation of a reliable phylogenetic tree of the *S. suis* core genome is impossible given the evidence of widespread recombination (Weinert *et al.* (9)). Nevertheless, it is tempting to speculate a role for the phase-variable Type I methyltransferase in the disease phenotype of *S. suis* given the likely acquisition of the Type I system at the origin of the globalized highly virulent zoonotic lineage in the 1920s (Weinert *et al.* (9)). However, an alternative scenario is that different genetic elements are responsible for the disease phenotype and that the Type I system is in syntenic linkage with these elements. The phase-variable Type III methyltransferase shows a stronger association with respiratory and non-clinical isolates suggesting a possible role for colonization (Table 4) but again, phylogenetic non-independence complicates this finding. Further sampling of *S. suis* for presence/absence of phase-variable Type I and the Type III methyltransferases may give us the necessary statistical power to see an association that remains robust after controlling for *S. suis* relatedness. As we always see the presence of GAGCA_(n)_ repeats in the genomes of our *S. suis* strain collection, but these are not always linked to the downstream *mod* gene due to the apparent requirement of the observed 119 bp ‘linker’ sequence we observed in the 41 strains containing a phase-variable Type III *mod* gene, it may be that acquisition of a phase-variable Type I locus leads to subsequent inactivation of the Type III *mod* gene by loss of the 119 bp ‘linker’ required for phase-variation of the Type III locus, but this would require subsequent investigation to confirm.

Our demonstration of the presence of several unique phase-variable methyltransferases in *S. suis* leads to the possibility of multiple phasevarions in this species. The presence of phasevarions complicates the development of vaccines and therapeutics, as the stably expressed protein profile of *S. suis* can only be defined by dissecting which genes are controlled by phasevarions in this species.

## Supplementary Material

Supplementary Data
