# Supplementary material for: Streptococcus suis contains multiple phase-variable methyltransferases that show a discrete lineage distribution
Source: Nucleic Acids Res. 2018 Oct 10;46(21):11466–76. doi: 10.1093/nar/gky913 (PMC6265453; doi:10.1093/nar/gky913)
Supplement: Supplementary Data [file gky913_supplemental_files.zip › Supplementary Figure 1.pdf]

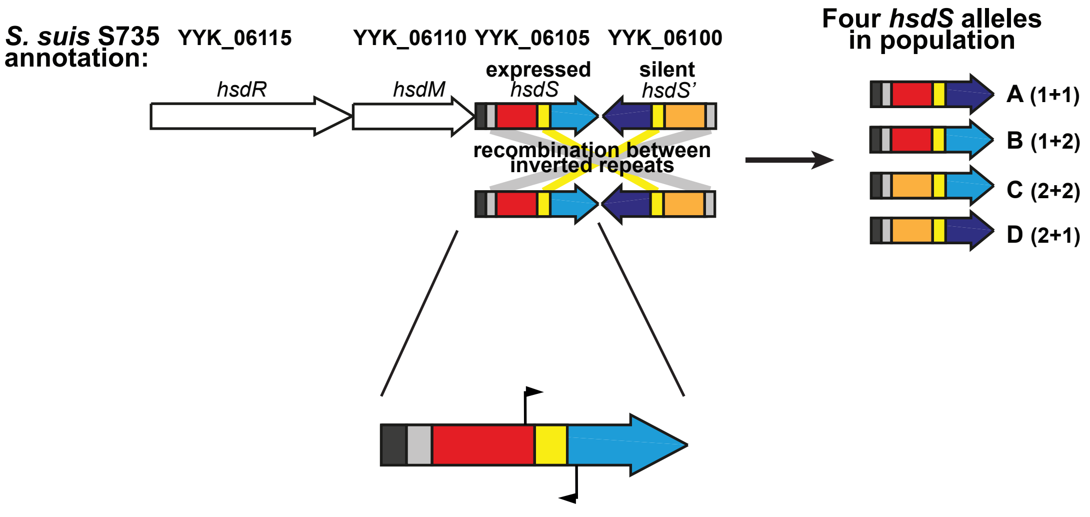

e.g., allele B in expressed hsdS locus

↓

primers specific for 5'-TRD-1 and 3'-TRD-2 = only give a product for allele B specific cDNA

↓

intensity of band proportional to amount of cDNA present

expected sizes - A = 364bp; B = 405bp; C = 413bp; D = 372bp

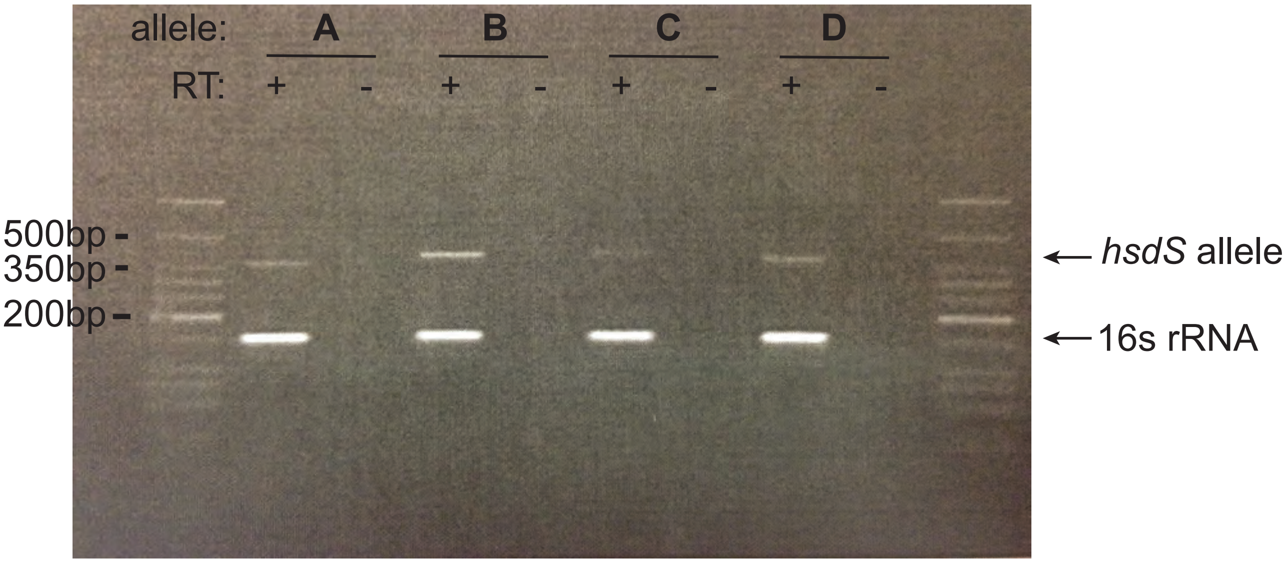

RT-16S-Ssu-F	ATGGACCTGCGTTGTATTAGC
RT-16S-Ssu-R	CATTGCCGAAGATTCCCTAC
RT-SsuT1-5'_1-F	GGT TTT GGA GAT ACA CTT CTC C
RT-SsuT1-5'_2-F	CCA TTC GTA GCA CAA ACT CAA C
RT-SsuT1-3'_1-R	GAA GAG TCC AAT TTC TCG GAA TC
RT-SsuT1-3'_2-R	GTA GCA CCA AGA ACT AGG AAG

Supplementary Figure 1 - semi-quantitative RT-PCR (sq-RT-PCR) to demonstrate that all four hsdS alleles are expressed in a population of S. suis strain S735. RNA was prepared from overnight grown *S. suis* strain S735 using Trizol reagent as described in Materials and Methods. cDNA was synthesised from this RNA, and the cDNA used as template in a PCR reaction using 16srRNA specific primers as a control, and one of four primer pairs specific for one *hsdS* allele - in the example shown, for allele B, the primer pair used was RT-SsuT1-5'_1-F and RT-SsuT1-3'_2-F - these primers are specific for 5' TRD 1 and 3' TRD 2, and will only give a product for allele B, which is consists of 5' TRD 1 and 3' TRD 2 (Figure 1). Using allele specific primer pairs, we demonstrate the presence of cDNA, and therefore mRNA, for each of the four encoded *hsdS* alleles, demonstrating that all four variants are expressed in the population of *S. suis* strain S735. Expected sizes each product - allele A = 364bp; allele B = 405bp; allele C = 413bp; allele D = 372bp; 16s rRNA specific primers serve as a control for the amount of cDNA in each PCR reaction.
